# Predictive Values of Urinary Interleukin 18 and Neutrophil Gelatinase-Associated Lipocalin for Delayed Graft Function Diagnosis in Kidney Transplantation

**Published:** 2016

**Authors:** Mojtaba Mojtahedzadeh, Farhad Etezadi, Javad Motaharinia, Amir Hossein Najafi Abrandabadi, Abdorasul mehrsai, Shadi Ziaie, Soheil Saadat

**Affiliations:** 1 *Faculty of Pharmacy, Tehran University of Medical Sciences, Tehran, Iran*; 2 *Dept. of Anesthesiology & Critical Care, Sina Hospital, Tehran University of Medical Sciences, Tehran, Iran*; 3 *Urology Research Center, Tehran University of Medical Sciences, Tehran, Iran*; 4 *Faculty of Pharmacy, Shahid Beheshty University of Medical Sciences, Tehran, Iran*; 5 *Trauma Research Center, Sina Hospital, Tehran University of Medical Sciences, Tehran, Iran*

**Keywords:** Kidney transplantation, Deceased donor, Delayed graft function, Interleukin 18, Neutrophil gelatinase-associated lipocalin

## Abstract

**Background:**

Delayed graft function is a main complication after deceased donor kidney transplantation that adversely affects graft outcome. Difficulties in prediction and early detection of delayed graft function have hindered the ability to perform proper therapeutic interventions. We investigated whether measuring urinary interleukin 18 and neutrophil gelatinase-associated lipocalin as markers of ischemia reperfusion injury could predict delayed graft function in deceased donor kidney transplant patients.

**Methods:**

We studied 69 patients undergoing kidney transplantation from deceased donor during early October 2013 to December 2014 at the Urology Research Center, Sina Hospital, Tehran University of Medical Sciences, Tehran, Iran. Serial urine samples at 2, 24, and 48 h after transplantation were analyzed for interleukin 18 and neutrophil gelatinase-associated lipocalin levels.

**Results:**

Thirteen patients (18.9%) developed delayed graft function. Urine interleukin 18 to urine creatinine ratio was significantly higher in patients with delayed graft function compared to those with non-delayed graft function, at 2 (*P*=0.003), 24 (*P*<0.001) and 48 h (*P*=0.018) points. The levels of neutrophil gelatinase-associated lipocalin to urine creatinine ratio were significantly higher in the group with delayed graft function at the 24 (*P*=0.004) and 48 h (*P*=0.015) points. The receiver–operating characteristic curve analysis suggested that both urinary biomarkers at 24 h after transplantation had better accuracies for prediction of delayed graft function. In multivariate analysis, only urinary interleukin 18 to urine creatinine ratio improved the ability of clinical model for predicting delayed graft function.

**Conclusion:**

Urinary interleukin 18 to urine creatinine ratio at 24 h post-transplantation, along with traditional markers such as relative fall in serum creatinine, urine output and other risk factors for delayed graft function, increased the ability to predict delayed graft function.

## Introduction

Kidney transplantation is a cardinal method and the most cost-effective treatment modality for patients with end-stage renal disease (1). Ischemia and reperfusion during kidney transplantation result in acute graft injury, presents as slow graft function (SGF), delayed graft function (DGF), or primary graft non-function based on the severity of damage (2).

According to the United States Renal Data System (USRDS) in 2009, the incidence of DGF in deceased donor kidney transplants ranges from 20% for standard criteria donors to 31% for extended criteria donors (3). DGF and SGF decrease graft survival, and increase the risk of graft acute rejection (4). 

Different definitions based on creatinine, urine volume, and the need for dialysis have been provided for DGF, the most common of which is the need for dialysis in the first week after transplantation. The dependence of DGF diagnosis on non-critical variables such as creatinine and medical decision-making on the need for dialysis has made DGF diagnosis a big challenge for kidney transplants to date. 

Although various scoring systems have been designed to predict DGF according to the donor and recipient parameters (5), replacing sensitive and specific surrogate markers for DGF can contribute to the prediction of DGF and subsequent necessary actions in due time (6). Interleukin 18 (IL-18) and neutrophil gelatinase-associated lipocalin (NGAL) have a pathogenic and protective role in kidney ischemia reperfusion injury, respectively (7). Urinary IL-18 and NGAL measurements in the early days after kidney transplantation can predict DGF (7). 

There is not enough information on which biomarker has a better predictive value, time of measurement, and cut-off value. In addition, previous studies have been conducted among different populations, in terms of the cause of brain death and duration of cold ischemia. Therefore, we aimed to evaluate the role of urinary biomarkers in predicting DGF, among patients undergoing kidney transplantation from a deceased donor.

## Materials and Methods

This prospective cohort study was carried out on adult patients undergoing first time kidney transplantation, from deceased donors during early October 2013 to December 2014 at the Urology Research Center, Sina Hospital, Tehran, Iran with the aim of evaluating the role of urinary biomarkers in predicting DGF. 

The study was approved by the Ethics Committee of Tehran University of Medical Sciences and the patients were enrolled after giving written informed consents. Patients with graft impairment caused by renal artery thrombosis or bleeding from the graft vascular anastomosis were excluded.

The primary endpoint was DGF. Delayed graft function was defined by need for dialysis within the first week after transplantation or when serum creatinine level decreased by less than 10% per day immediately after transplantation. To measure the levels of urinary biomarkers at 2, 24, and 48 h after transplantation, 5 ml of urine samples were collected directly from the patients’ catheters. The urine samples were centrifuged at 5000 × g at room temperature and stored at -80 °C. The IL-18 and NGAL levels were measured in a blind approach to patients’ information using the Human IL-18 ELISA Kit and Human NGAL ELISA Kit (Shanghai crystal day biotech, China). The inter-assay variability coefficient was less than 12% for the biomarkers. Urinary biomarkers were normalized to urinary creatinine concentration, in order to account for the differences in glomerular filtration rate and resultant urinary flow.

The immunosuppressive regimen involved induction with daily anti-thymocyte globulin (ATG), at a dose of 1 mg/kg for 5 d, followed by triple-drug immunosuppressive therapy. All patients received methylprednisolone 500 mg intraoperatively, 250 and 100 mg on the second and third day post-transplant, then converted to prednisone and tapered to 5 mg daily after several months. Mycophenolate mofetil was administered at a dose of 1g twice daily, starting the day of transplantation. Oral administration of cyclosporine started after transplantation at a dose of 6 mg/kg/day, divided twice daily.

The clinical data were collected from the patients’ records and the National Kidney Transplantation Registry database. Recipient variables included age, sex, body mass index, cause of end-stage renal disease, time on dialysis pre-transplantation, hemodialysis time before transplantation procedure, daily serum creatinine until discharge and daily urine output within the first week after transplantation. Donor variables included age, sex, cause of brain death, serum creatinine and duration of cold ischemia.

Patient demographic information and baseline characteristics were summarized using descriptive statistics. The recipient, donor, and transplant parameters were compared using unpaired two-sample *t* tests or cross-tables and Fisher’s exact test. If the statistical assumptions for parametric analysis of interval variables were not met, Mann–Whitney’s two-independent samples test was used. The biomarker levels between patients with DGF, and non-DGF were compared using unpaired two-sample *t* tests. The receiver–operating characteristic (ROC) curve analysis was performed to compare the accuracy of IL-18, and NGAL at each time point, for the prediction of DGF. The associations between biomarkers and DGF were evaluated in a multivariate logistic analysis while adjusting the variables selected by univariate analysis and risk factor for DGF. Multivariate stepwise multiple logistic regression and integrated discrimination improvement (IDI) analyses were performed to assess added values of urinary biomarkers for predicting DGF. A value of *P*<0.05 was considered statistically significant. Analyses were conducted using the SPSS version 19 (Chicago, IL, USA). IDI has been calculated using the package PredictABEL in R (R version 3.2.0. R Foundation for Statistical Computing, Vienna, Austria).

## Results

Seventy-five (75) patients were enrolled in this study. Of these, six patients were excluded due to surgical site/anatomical complications. The characteristics of donors and recipients, as well as findings in patients DGF and non-DGF are given in Table 1. The donors and recipients’ characteristics were not significantly different between the 2 groups of patients. However, the duration of cold ischemia was longer in the patients with DGF. All the study patients received kidneys from standard criteria donors, all were hemodialyzed, except for 2 patients. The DGF rate in the patients was 18.9% (n = 13). Dialysis was started at a mean 12 h (range, 8 – 38 h) after transplantation and the main cause of dialysis in patients with DGF was azotemia. 

**Table 1:** Clinical characteristics of patients with different graft function

Recipient characteristics Total (n = 69)			Non-DGF (n = 56)	DGF (n = 13)		*P* value
	Age (yr)		42 (12)^a^		43 (13)	0.76
	Sex (% male)		82.2^a^		69.2	0.34
	BMI (kg/m^2^)		24.2 (4.5)		25.7 (4.0)	0.27
	Cause of end-stage renal disease					
		Diabetes	27		25.5	0.33
		Hypertension	32		29	
		Obstructive uropathy	10		15.5	
		Infection	5.5		10.5	
		Polycystic kidney	5.5		8	
		Glomerulonephritis	7		2.5	
		Others	13		5	
	Time on pre-transplant dialysis (month)b		16 (60)		18 (87)	0.72
	Last dialysis session before transplant (day)b		1 (2)		1 (2)	0.79
	Serum creatinine before transplant (mg/dl)		6.54 (1.90)		7.2 (1.82)	0.25
Donor characteristics						
	Age (yr)		31 (12)		35 (14)	0.29
	Sex (% male)		70		56	0.32
	Serum creatinine (mg/dl)		1.25 (0.35)		1.30 (0.52)	0.57
	Cause of brain death					
		CVA	19		15.4	0.1
		Head trauma	62.3		61.5	
		Post CPR	7.9		0	
		Drug Intoxication	5.7		7.7	
		Brain tumor	5.1		15.4	
	Cold ischemic time (h)		3.97 (0.88)		4.64 (1.43)	0.035
	Warm ischemic time (min)		45 (7)		43 (9)	0.5
Study findings						
	Urinary NGAL (ng/mg Cr)					
		2h post-transplant	8593 (4927)		8883 (4578)	0.065
		24h post-transplant	8666 (5432)		14247 (6137)	0.004
		48h post-transplant	10359 (5941)		11923 (6483)	0.015
	Urinary IL-18 (pg/mg Cr)					
		2h post-transplant	1737 (1258)		3847 (2349)	0.003
		24h post-transplant	1942 (1113)		3168 (2077)	< 0.001
		48h post-transplant	2132 (1171)		2725 (1883)	0.018
	Relative fall in serum creatinine at 24 h post-transplant		0.29 (0.13)		0.16 (0.09)	0.002
	First 24 h post-transplant UOP (ml)		7943 (2819)		2775 (553)	<0.001

DGF, delayed graft function; BMI, body mass index; CVA, cerebrovascular accident; CPR, cardiopulmonary resuscitation; NGAL, neutrophil gelatinase-associate lipocalin; IL-18, interleukin 18; Cr, creatinine; UOP, urine output.

a Data presented as mean (SD) or percentage.

b Data presented as median (range)

In this study, urine was selected as a source for determining graft function biomarkers, although it was not possible to determine the levels of urinary biomarkers before transplantation, since the majority of the patients were anuric or oliguric. Two patients with DGF had post transplantation anuria so were excluded from multivariate analysis because of the absence of urinary sample.

The mean urine IL-18 to urine creatinine ratios (IL-18/Cr) were significantly higher in patients with DGF compared to those with non-DGF, at 2 (*P*=0.003), 24 (*P* <0.001) and 48 h (*P*=0.018) after transplantation. The levels of NGAL to urine creatinine ratio (NGAL/Cr) were significantly higher in the group with DGF at the 24 (*P*=0.004) and 48 h (*P*=0.015) points (Table 1). From the analysis of covariance, cold ischemia time, and other risk factors for DGF including donor age, donor serum creatinine, recipient age, recipient body mass index, time on dialysis pre-transplantation did not affect the urinary NGAL/Cr and IL-18/Cr. 

ROC curve analyses were performed to determine which biomarker and time point was a better predictor of DGF. Table 2 shows the area under the curve (AUC) of each biomarker. From the different times of measurement for urinary IL-18/Cr and NGAL/Cr, the greatest AUC was related to 24 after transplantation. The cut-off point of 2500 pg/mg Cr for IL-18 had a sensitivity of 89%, specificity of 81%, and diagnostic odds ratio (DOR) of 9.9 (98% CI 2.0 – 49.2). A cut-off point of 8700 ng/mg Cr for the 24 h post-transplant NGAL, produced an 83% sensitivity and specificity of 78%. The DOR for this cut off point was 5.9 (95% CI 1.2 – 29.1). For comparison, ROC analyses was also performed for the relative fall in serum creatinine at 24 h post-transplant and urine output (UOP) in the first 24 h post-transplant. These variables had AUC comparable to the 24 h post-transplant NGAL/Cr and IL-18/Cr (Table 2). In order to determine urinary NGAL/Cr and IL-18/Cr as independent predictors of DGF, multivariable analyses were performed with the risk factors of DGF. Only, urinary IL-18/Cr was significant independent predictor of DGF (Table 3).

**Table 2:** Area under the receiver-operating curves for DGF prediction

			AUC	CI 95%			*p* value
Urinary NGAL								
	2h post-transplant	0.674			0.489 – 0.848	0.060		
	24h post-transplant	0.790			0.633 - 0.930	0.002		
	48h post-transplant	0.814			0.649 - 0.973	0.001		
Urinary IL-18								
	2h post-transplant	0.800			0.634 - 0.938	0.001		
	24h post-transplant	0.841			0.708 – 0.973	< 0.001		
	48h post-transplant	0.729			0.548 - 0.887	0.015		
**Relative fall in serum creatinine at 24 h post-transplant**		0.821			0.712 – 0.930	< 0.001		
**First 24 h post-transplant UOP**		0.782			0.629 – 0.934	0.002		

AUC, area under the curve; CI, confidence interval; NGAL, neutrophil gelatinase-associate lipocalin; IL-18, interleukin 18; UOP, urine output.

**Table 3:** Multivariate logistic regression analysis of risk factors and urinary biomarkers for DGF

Variable	Odds ratio (95% CI)	*P* value
**Recipient age**	1.004 (0.921 – 1.094)	0.935
**Recipient BMI**	0.939 (0.732 – 1.205)	0.62
**Donor age**	0.989 (0.907 – 1.078)	0.8
**Donor serum creatinine**	1.375 (0.53 – 35.358)	0.85
**Cold ischemia time**	1.820 (0.781 – 4.240)	0.168
**First 24 h post-transplant UOP**	1.000 (1.000 – 1.000)	0.68
**Relative fall in serum creatinine at 24 h post-transplant**	0.005 (0.000 – 217.916)	0.327
**IL-18 at 24 h post-transplant**	1.068 (1.008 – 1.131)	0.025
**NGAL at 24 h post-transplant**	1.013 (0.998 – 1.028)	0.087

DGF, delayed graft function; CI, confidence interval; BMI, body mass index; UOP, urine output, IL-18, interleukin 18; NGAL, neutrophil gelatinase -associated lipocalin.

Logistic regression models and integrated discrimination improvement (IDI) analysis were used to assess the added value of 24 h post-transplant urinary IL-18/Cr and NGAL/Cr to clinical variables in prediction of DGF. First, variables including the first 24 h UOP, the relative fall in serum creatinine at 24 h post-transplant, and standard risk factors for DGF (recipient age, body mass index, cold ischemia time, donor age, donor serum creatinine) entered as a clinical model (Table 4). Next, IL-18/Cr and NGAL/Cr were added in a stepwise manner to the clinical model. Addition of IL-18/Cr improved the prediction of DGF, as seen by the improvement in AUC with an absolute IDI = 0.075. However, when NGAL/Cr was added to the clinical or combined models (Clinical model + IL-18), accuracy of the prediction models improved a little (Table 4).

**Table 4:** Receiver operating characteristic curve and integrated discrimination improvement analysis of the addition of each or the combination of 24 h post-transplant urinary NGAL and IL-18 to the clinical model for DGF

DGF prediction models		AUC (95% CI)	IDI	*P* value
**Clinical model**		0.802 (0.667 – 0.937)		
	+ NGAL	0.833 (0.721 – 0.946)	0.0356	0.023
	+ IL-18	0.871 (0.750 – 1.000)	0.0757	0.003
	+ IL-18 + NGAL	0.905 (0.815 – 1.000)	0.0796	< 0.001

The clinical model included relative fall in serum creatinine, urine output, and recipient age, body mass index, cold ischemia time, donor age, donor serum creatinine. IL-18, interleukin 18; NGAL, neutrophil gelatinase – associated lipocalin; DGF, delayed graft function; AUC, area under the curve; CI, confidence interval; IDI, integrated discrimination improvement

## Discussion

Delayed graft function is associated with worse long-term graft outcome (4). Timely detection or prediction of DGF occurrence reduces DGF adverse impacts on the graft through opportune therapeutic interventions (4). A variety of clinical parameters have been proposed for the prediction of DGF based on the preoperative risks, but unfortunately no objective and reliable markers exist for early detection of DGF. Recently, urinary IL-18 and NGAL have been proposed as early biomarkers for DGF (7). 

In this research, the incidence of DGF was 18.9%, but according to USRDS in 2009, the rate in patients undergoing transplants from deceased donors has been between 20 and 31% (3). The duration of cold ischemia is one of the most important risk factors for DGF, representing a 10% increase in DGF, for every 6-hour increment (8). In this study, cold ischemia time was between 3 and 6 h and it seems the shorter duration of cold ischemia is one of the reasons for the low rate of DGF. Additionally, low age and the leading cause of brain death in our kidney donors (driving accidents), are involved in reducing the DGF rate (9). It is known that with the aging of donors, DGF rate increases in such a way that its rate has been reported to be 15% and 40% for donors aged between 20 and 65 yr, respectively (8). According to United Network for Organ Sharing, the DGF rates of donors who died by driving accident and cerebrovascular events are 18 and 30%, respectively (8). Given the differences in the durations of cold ischemia and kidney donors between the study's sample community and other populations, Our study was performed to determine if the urinary biomarkers are capable of predicting DGF in this study's patient population. 

Based on ROC analysis, urinary IL-18/Cr and NGAL/Cr were better at discriminating DGF from non-DGF at 24 h. When AUCs of urinary NGAL/Cr and IL-18/Cr for 24 h in our study were compared with the results of the previous studies (10-13), urinary NGAL/Cr and IL-18/Cr of 24 h showed comparable accuracies for predicting DGF. However, Parikh et al. reported a better AUC for NGAL/Cr and IL-18/Cr in prediction of DGF, but the patient population was consisted of both living and deceased donor kidney transplantation (14). In our study, a relative fall in serum creatinine had comparable AUC to IL-18/Cr, but had higher AUC compared to NGAL/Cr. In addition, UOP had modest diagnostic performance. Multivariate analysis revealed IL-18/Cr, as independent predictors of DGF but did not support the use of relative fall in serum creatinine and UOP. This consistent with previous studies that the absolute and relative fall in serum creatinine on the first postoperative day, did not predict DGF (12, 13). 

Adding IL-18/Cr, significantly improved the prediction of the clinical model for the diagnosis of DGF, but NGAL/Cr did not improve the prediction ability of the model. This is in contrast to previous studies where the 24 h urinary NGAL/Cr, increased the ability of clinical variables to predict DGF (10, 12-15). Perhaps, in this study, the short duration of cold ischemia is one of the reasons for the lack of superior prediction ability of urinary NGAL/Cr. Previous findings in cases of acute kidney injury (AKI) due to other causes show that urinary NGAL alone is an imperfect tool in the early prediction of AKI (16). The AUC measured in previous studies (0.693-0.800), and urinary NGAL, have no advantage compared to the traditional marker (16). The relative performance of urinary NGAL and IL-18 for prediction of DGF is time-dependent (17). They measured urinary IL-18 and NGAL at 4 and 12 h after transplantation, in deceased donor kidney transplant patients with mean cold ischemia time of 10 h. Although the AUC for urinary IL-18/Cr and NGAL/Cr at 4 h after transplantation were 0.7 and 0.77, respectively, the IDI analysis showed that IL-18 increased ability to predict DGF, but NGAL lacks this ability (17). 

The small sample size is the main limitation of this study. The DGF was determined in the first week after transplantation based on the need for dialysis. However, the clinical judgment of practitioners on the need for dialysis may overestimate the rate of DGF and result in a possible bias.

## Conclusion

A measure of IL-18/Cr at 24 h post-transplantation, along with traditional markers such as relative fall in serum creatinine, UOP and other risk factors involved in DGF, increased the ability to predict DGF. Measuring urinary NGAL/Cr at 24 h post-transplantation did not help in the diagnosis of DGF. In this study, the concurrent measurement of urinary IL-18/Cr and NGAL/Cr than IL-18/Cr alone, did not lead to an increase in the predictive power of DGF. 
